# Electrochemical Characterization of Neurotransmitters in a Single Submicron Droplet

**DOI:** 10.3390/bios14020102

**Published:** 2024-02-17

**Authors:** Heekyung Park, Jun Hui Park

**Affiliations:** Department of Chemistry, Chungbuk National University, Cheongju 28644, Republic of Korea

**Keywords:** dopamine, redox reactions, nanoelectrochemistry, recollision, single entity, nanoreactor

## Abstract

Single-entity electrochemistry, which employs electrolysis during the collision of single particles on ultramicroelectrodes, has witnessed significant advancements in recent years, enabling the observation and characterization of individual particles. Information on a single aqueous droplet (e.g., size) can also be studied based on the redox species contained therein. Dopamine, a redox-active neurotransmitter, is usually present in intracellular vesicles. Similarly, in the current study, the electrochemical properties of neurotransmitters in submicron droplets were investigated. Because dopamine oxidation is accompanied by proton transfer, unique electrochemical properties of dopamine were observed in the droplet. We also investigated the electrochemical properties of the adsorbed droplets containing DA and the detection of oxidized dopamine by the recollision phenomenon.

## 1. Introduction

Dopamine (DA), a neurotransmitter extensively studied for its multifaceted roles in various diseases, is the focus of this study [1,2]. Abnormal concentrations of DA in biological systems can manifest in various diseases (e.g., Parkinson’s), making it a critical diagnostic indicator [3]. The DA detection method has been studied for decades for selective analysis, excluding interfering species (e.g., ascorbic acid and uric acid) owing to redox potential overlap [4,5,6]. Although the precise electrochemical mechanism underlying the oxidation of DA through proton-coupled electron transfer (PCET) has not been completely elucidated [7], several methods have been developed to enhance detection sensitivity [8,9]. Over the years, researchers have modified ultramicroelectrodes (UMEs) to improve sensitivity and selectivity, as detailed in Table 1. However, in a real system, DA is contained inside a synaptic vesicle; therefore, a detection system for dopamine inside a single vesicle is required [10].

Single-entity electrochemistry, a technique designed to measure the characteristics of individual particles that has rarely been studied in macro-sized electrode systems because the loading of a single particle is almost impossible, has undergone significant advancements through the use of detection methods employing the collisional contact of a single particle on UMEs [16,17]. Over the last decade, various single entities, including metal nanoparticles [18,19,20,21,22,23,24,25], biomaterials [26,27], nanobubbles [28,29], vesicles [30,31], and droplets [32,33,34,35,36,37,38,39,40], have been analyzed electrochemically at the single-entity level. The entity size can be determined by measuring the amperometric current response obtained from a single collision event [18,39]. This technique also provides particle information, such as the concentration of redox species contained inside the entity, the contact area [41,42], the thermodynamic constant, and the kinetic constant [43,44,45]. Notably, single liquid droplets such as water-in-oil emulsion droplets can serve as convenient platforms for studying reactions in confined spaces. Water-in-oil droplets can be easily adjusted with different types of redox species, concentrations of redox species, ionic compositions, and surfactant molecules to mimic the nature of the system of vesicles. The electrochemical properties obtained from this platform are also helpful for understanding the nanoscale electrochemistry of a single vesicle.

In this study, we investigated the unique electrochemical properties of dopamine in a single submicron-sized aqueous droplet based on collisional contact with an ultramicroelectrode. Dopamine in a single droplet was adsorbed on the electrode through a unique electrochemical response arising from the PCET mechanism of dopamine oxidation.

**Scheme 1:**
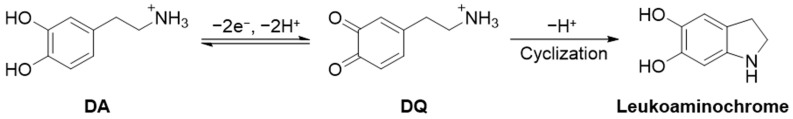
Simplified dopamine electrochemical oxidation and further chemical reaction mechanism.

The electrochemical oxidation of DA was combined with proton transfer, as shown in Scheme 1. DA is a molecule with an ammonium group in catechol that can be quasi-reversibly oxidized from DA to DQ [46]. While further chemical reactions (i.e., cyclization) decrease reversibility, the local pH may decrease because of the generation of protons during the dopamine electrochemical oxidation reaction. This is particularly evident in bulk electrolysis, at least for a short time until the produced proton diffuses away from the electrode. When the reaction volume is extremely small, such as in submicron droplets, the produced protons change the overall pH of the droplet, resulting in a reduction in and potential shift of the dopaquinone. This also implies the suppression of the cyclization reaction of DQ to produce leukoaminochromes inside a single entity (e.g., vesicles). We also investigated the electrochemical properties of a detached droplet containing oxidized dopamine via the recollision phenomenon [47].

## 2. Materials and Methods

### 2.1. Chemicals

All organic solvents (1,2-dichloroethane, DCE, 99.5%; anhydrous ethanol, 99.5%; acetone, 99.7%; acetonitrile, ACN, 99.7%; *n*-hexane, 96.0%; toluene, 99.8%; and 2-propanol, IPA, 99.5%) were purchased from Samchun Pure Chemicals (Pyeongtaek, Republic of Korea). All aqueous solutions were prepared using purified water (Milli-Q, 18.2 MΩ·cm), and all organic solutions within the electrochemical cell were made by DCE except for ACN, which was used as a stock solution of a non-aqueous reference electrode. Magnesium sulfate anhydrous (99.0%) was purchased as an aqueous electrolyte from Daejung Chemicals (Siheung, Republic of Korea). Dopamine hydrochloride (99%), potassium chloride (99.0%) (as an electrolyte for a stock solution of an aqueous reference electrode), tetrabutylammonium hexafluorophosphate (TBAPF_6_, 98.0%) (as the organic electrolyte), and ferrocenemethanol (97%) (for checking the UME surface area) were purchased from Sigma-Aldrich (St. Louis, MO, USA). Dioctyl sulfosuccinate sodium salt (AOT) (as the surfactant) and silver nitrate (99.9%) (as the electrolyte for stock solution of a non-aqueous reference electrode) were purchased from Alfa Aesar (Ward Hill, MA, USA). All the reagents were used as received.

### 2.2. Materials

Borosilicate glass capillary (1.5 mm O.D. × 0.75 mm I.D.) as a UME body was purchased from Sutter. Pt wire (99.997%, 2 mm diameter), used as the counter electrode, was purchased from Alfa Aesar. CHI112 used as a non-aqueous reference electrode, was purchased from CHI Instruments (Austin, TX, USA). Metal wires (99.9% gold and platinum, respectively) with a diameter of 10 μm were purchased from Goodfellow (Devon, PA, USA). Silicon carbide abrasive sandpapers (400, 1000, 1200, 2400, and 4000 grit) for the mirror surface of the UMEs were purchased from R&B Co., Ltd. (Daejeon, Republic of Korea).

### 2.3. Instruments

Electrochemical experiments were performed using a CHI 750 potentiostat (CHI Instruments, Austin, TX, USA) with 3-electrode systems in a faradaic cage. Inverse emulsions were prepared using a Q700 ultrasonic processor (Qsonica, Newtown, CT, USA). Dynamic light scattering (DLS) was used to measure particle size using a NanoBrook 90Plus particle size analyzer (Brookhaven Instruments Corporation, Holtsville, NY, USA). Two types of reference electrodes were used in two different solvents. In aqueous solutions, a Ag/AgCl reference electrode (0.197 V vs. NHE) containing saturated KCl was used, whereas, in organic solvents, a Ag/Ag^+^ reference electrode (0.541 V vs. NHE) was prepared using a silver wire and a membrane containing 10 mM AgNO_3_ and 100 mM TBAPF_6_ in ACN.

### 2.4. Preparation of Metal UMEs

The UME was prepared using a developed laboratory method. Briefly, a single metal wire was placed in a borosilicate glass capillary and sonicated in hexane, toluene, IPA, ethanol, and water. The UME was polished from 400 to 4000 grit using a silicon carbide abrasive sandpaper to a mirror finish. The surface area of the UME was determined using standard redox cyclic voltammetry in a ferrocenemethanol solution. Before each electrochemical experiment, all UMEs were polished using 4000-grit SiC sandpaper.

### 2.5. Preparation of Inverse Emulsion

The water-in-oil inverse emulsion, an aqueous DA droplet, was prepared as follows: Briefly, 50.0 mM of DA and MgSO_4_ were dissolved in distilled water, and 200 μL of this aqueous solution was added to 5.0 mL of DCE with 1 mM AOT. The mixture was then stirred using an ultrasonicator (700 W, amplitude 30%) in pulse mode (3 s on and 7 s off for 10 repeated cycles). The diameter of the droplets was 0.5–3.5 µm from DLS intensity.

## 3. Results and Discussion

**Scheme 2:**
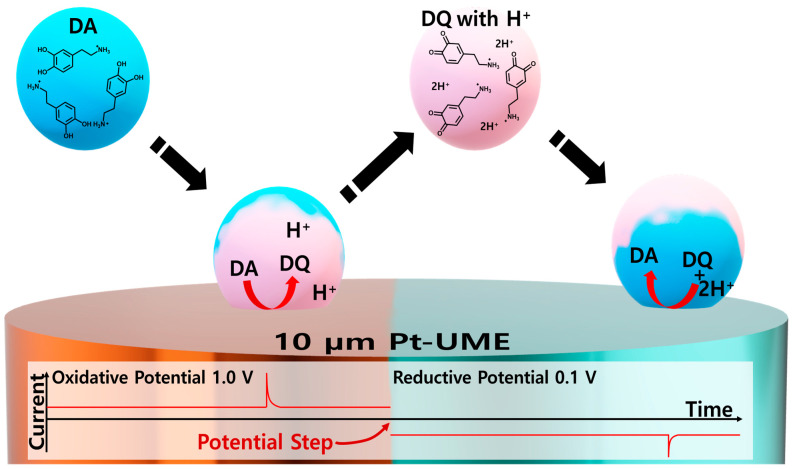
Schematic of collision and recollision of the droplet and relevant reactions proceeding within the droplet.

Although sensitive and selective detection of DA in the presence of the interfering species is possible using a screen-printed electrode (SPE) or glassy carbon (GC) electrode, detecting dopamine in attoliter droplets using an SPE or GC electrode is challenging due to the large capacitive current, which is proportional to the electrode area [48,49,50,51]. With UME having a micron-scale electroactive area, the dopamine in the attoliter vesicle can be detected. Additionally, a small electroactive area minimizes the possibility of simultaneous collisions, enabling the analysis of each collision individually. As shown Scheme 2, a water-in-oil droplet was prepared using a surfactant for long-term stability to produce dopamine confined to nanoscale entities such as synaptic vesicles. Dioctyl sulfosuccinate sodium salt (AOT) was used as the stabilizing agent for the water-in-oil droplets. An aqueous droplet containing DA in organic solvent was electrochemically monitored at the oxidizing electrode potential. The collisional contact of a DA droplet can exhibit a current spike because the oxidative reaction starts instantly at the moment of contact. After oxidation of the dopamine in the droplet, it may detach from the electrode surface. The detached droplets should contain hydrophilic DQ. If the electrode potential shifts to the reductive potential after observing the oxidative spike current, the electrode can again detect DQ in the droplet when the droplet collides with the electrode, generating a reductive spike current.

DQ can also undergo chemical reactions inside the droplets, as shown in Scheme 1. Furthermore, it should be noted that two protons (H^+^) are required to reduce the DQ to DA. The proposed single-entity-based detection method for DQ is a powerful platform for investigating reaction products (or intermediates) by isolating the products of the reaction inside a droplet.

A potential sweep experiment was conducted to determine the optimal potential for oxidative and reductive detection of a single droplet. A supporting electrolyte (e.g., MgSO_4_) was chosen to enhance the electrochemical reaction by decreasing the overpotential and increasing droplet stability [52].

**Figure 1:**
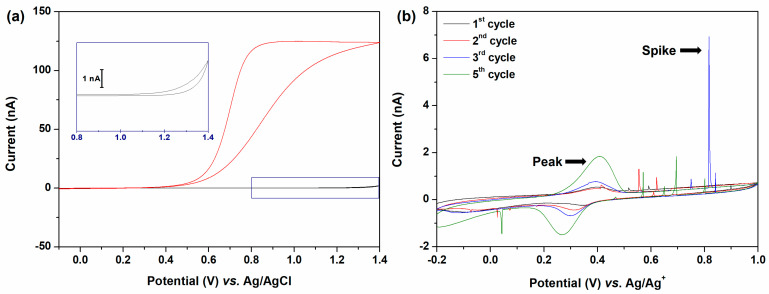
(**a**) Cyclic voltammograms recorded in a solution containing 50 mM MgSO_4_ in H_2_O using a 10 µm Pt UME. Each voltammogram represents the presence of 50 mM DA (red) and absence (black). The inset image provides an enlarged view of the black line. (**b**) Consecutive cyclic voltammograms were obtained in a solution containing 100 mM TBAPF_6_ in DCE with a 10 µm Pt UME after injecting 1 mL of emulsion. The emulsion contains 50 mM DA, 50 mM MgSO_4_, and 1 mM AOT. The black line corresponds to the first cycle, with the peaks of voltammograms increasing over time. All CV scan rates were 50 mV/s.

In Figure 1a, the cyclic voltammogram of DA is compared with that of an electrolyte solution. At the first positive sweep, the oxidative current increased, and then a plateau current was observed from 0.8 V (vs. Ag/AgCl), which is known as a steady-state current coming from the radial diffusion of the redox species. However, in the backward sweep, a plateau current is not observed, which is attributed to the reaction product (i.e., H^+^) that can change the local pH adjacent to the electrode. The redox potential can shift depending on the pH in a proton-involved electrochemical reaction. According to the Nernst equation, as shown in Equation (1), the redox potential of *DQ* ($E_{DQ}$) shifts in the positive direction under more acidic conditions. The potential difference between pH 0 and 7 is ca. 0.41 V at room temperature (Equation (1)) [53].

(1) $$E_{DQ}=E_{DQ}^{0^{\prime }}-\frac{RT}{nF}\ln\frac{C_{DA}}{C_{DQ}\times C_{H^{+}}^{2}}$$

(2) $$E_{DQ}=E_{DQ}^{0^{\prime }}-0.0296V\times \log\frac{C_{DA}}{C_{DQ}}-0.0592V\times pH$$

The $E_{DQ}$ is the observed potential (i.e., oxidation potential), R is the gas constant (8.314 J mol^−1^ K^−1^), F is the Faraday constant (96,485 C/mol) and $C_{i}$ is the concentration of species i. Equation (2) is presented while the temperature is 298.15 K (25 °C).

The oxidative current increased again in the background electrolyte solution when the potential exceeded 1.2 V (vs. Ag/AgCl). This was attributed to oxidation of the solvent (i.e., H_2_O). However, in the DA solution, the oxidation of the solvent was not observed at the same potential of 1.2 V, which was attributed to the local pH decrease.

As shown in Figure 1b, consecutive cyclic voltammetry measurements were conducted in an organic electrolyte solution with aqueous droplets containing 50 mM DA and 50 mM MgSO_4_ using a Pt UME electrode. The potential sweep was started from −0.2 V vs. Ag/Ag^+^ to 1.0 V to confirm that the DQ droplet had been generated by the collisional contact of the DA droplet with the electrode where oxidative potential was applied.

When the electrode potential was over 0.55 V vs. Ag/Ag^+^, the anodic collision spikes were observed owing to the electro-oxidation of DA to DQ. Subsequently, a cathodic collision spike was observed at 0.045 V vs. Ag/Ag^+^, indicating that the aqueous droplets containing reducible species (i.e., DQ) collided with the electrode. Reducible species are produced by prior oxidative collisions. The detection of the DQ droplet as a cathodic spiky current implied that some of the DA droplets colliding with the electrode detached from the electrode surface.

During consecutive cyclic voltammetry cycles, spike and peak currents occurred. The redox peak current provides significant information regarding the involvement of protons in the electron transfer of the DA/DQ redox couple.

Conversely, when a colliding droplet is adsorbed at the electrode, a redox peak current is observed, as in the electrochemistry of bulk electrode systems, where planar diffusion prevails. After the collision of the droplets indicated by the spike current, the droplets remained on the electrode, as indicated by the appearance of a peak current at 0.35 V (vs. Ag/Ag^+^) in Figure 1b. Multiple collision events were observed during voltammetry cycles. Accordingly, the peak current gradually increases owing to the accumulation of droplets on the surface of the electrode. The oxidation and reduction peak potentials exhibited distinct tendencies along with the increased peak current. Because dopamine oxidation produces two protons, as well as dopaquinone, the concentration of protons increases after a positive potential sweep. Considering the merging of a dopamine droplet with a dopaquinone droplet on the surface, the protons inside the droplet were partially diluted. Therefore, the cathodic peak potential shifted in the negative direction. During the subsequent negative sweep, the released proton is stored in dopamine. Therefore, the anodic peak potentials remain nearly unchanged.

In addition, DQ undergoes further chemical reactions (i.e., cyclizations). Based on previous studies, an increase in proton concentration can suppress the deprotonation of a Michael-type reaction from DQ to leukoaminochrome [46]. Therefore, the generated protons chemically stabilized DQ. In bulk solution, DQ tends to undergo cyclization to leukoaminochrome without assistance from the buffer solution. However, DQ within single entities was preserved owing to the cogenerated protons resulting from the oxidation of DA. This allowed us to obtain relatively more reversible electrochemistry of DA/DQ in single entities than in the bulk solution.

**Figure 2:**
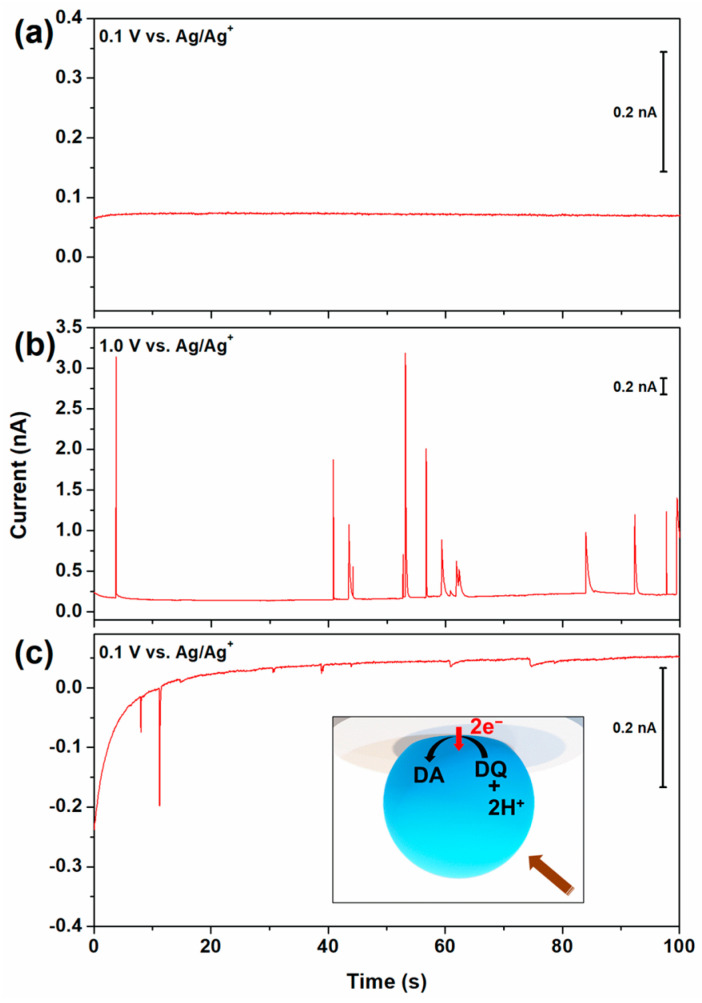
I–t amperometric curves of 100 mM TBAPF_6_ in DCE after injecting 1 mL of emulsion containing 50 mM of DA, MgSO_4_, and 1 mM of AOT at 10 µm Pt UME. The applied potentials are (**a**) 0.1 V vs. Ag/Ag^+^ for 100 s, (**b**) 1.0 V for 100 s, and (**c**) 0.1 V for 100 s immediately after applying the previous potential, respectively. The inserted image illustrates the electrochemical reduction of DQ in a simplified scheme.

To precisely measure the single-droplet information, constant potentials were applied to the electrode rather than using the potential sweep method. Sequential measurements at constant potentials were performed. Figure 2 shows the double-potential step chronoamperometry, alternating between reductive (0.1 V vs. Ag/Ag^+^), oxidative (1.0 V), and reductive (0.1 V) potentials to quantitatively analyze the anodic and cathodic current spikes. No spike signal was observed when a reduction potential was applied after the fresh DA droplets were injected. This observation confirmed that the DA droplets did not contain reducible species. Therefore, it can be suggested that the anodic collision spikes in Figure 2b correspond to DA oxidation, and the cathodic collision spikes in Figure 2c correspond to DQ reduction.

As indicated in Scheme 1, two protons are required to reduce DQ to DA. The cathodic spike currents indicate that protons may persist within the droplet. In single-entity electrochemistry, charge neutrality inside a droplet should always be maintained [37,54]. Upon collision with a droplet, which leads to proton generation as a side product, the internal charge balance within the droplet is effectively maintained through the transfer of cations from water to DCE or the transfer of anions from DCE to water, providing charge neutrality within the droplet. The Gibbs transfer energy of an ion from aqueous to DCE ($∆G_{tr,\;ion}^{0,\;water\to DCE}$) is higher for more hydrophobic ion molecules, and this can be considered an indicator of hydrophilicity. The Gibbs ion transfer energies for all ions present in the droplet are listed in Table 2. According to the Gibbs transfer energies shown in Table 2, it is more probable that PF_6_^−^ anions dissolved in the DCE transfer into the aqueous droplet than it is that H^+^ cations transferred out of the droplet to balance the charge neutrality.

Therefore, dopamine oxidation inside the droplet was not limited by charge imbalance because of the sufficient concentration of the hydrophobic electrolyte in the DCE solution. The charge quantity of colliding single droplets was determined by integrating the current spikes. The sizes of the individual droplets were estimated based on the extent of the redox reaction occurring inside the droplets. To simplify the calculations, it was assumed that the droplet containing DA was spherical, and a two-electron reaction was considered during complete oxidation. The droplet size was calculated using Equation (3), which was derived from Faraday’s Law.

(3) $$d_{droplet,DA}=\sqrt[{3}]{\frac{3Q_{DA}}{\pi FC_{DA}}}$$

where *Q*_DA_ is the integrated charge, *F* is the Faraday constant (96,485 C/mol), and *C*_DA_ is the DA concentration in an aqueous droplet.

**Figure 3:**
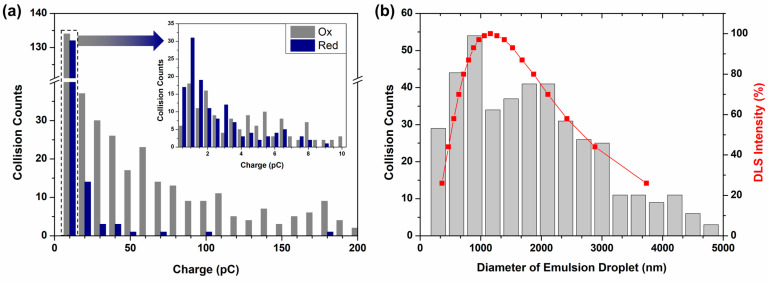
(**a**) Number of oxidative (gray) and reductive (blue) collision events classified according to the magnitude of the reacted charge. The inset shows a finer division of 0 to 10 pC. (**b**) Comparison of calculated data vs. DLS data. The gray bars were calculated from collision spikes obtained from I–t curves using Equation (3). The red solid line represents DLS intensity.

Figure 3a shows the distribution of spike charges obtained from double-potential step voltammetry, which comprised 430 oxidative current spikes and 157 reductive current spikes. The observed reductive collision frequency in Figure 3a was lower than the oxidative collision frequency. The frequency difference could be attributed to the considerably smaller number of DQ droplets generated by the collision compared with the DA droplets. The average charge values of oxidative and reductive collisions were 129.49 and 8.33 pC, respectively; the average charge ratio, Q_Ox_/Q_Red_, was 15.5. The relatively smaller charge of the DQ droplet may be attributed to the chemical reaction that proceeded in the droplet until it collided with the UME, although cyclization was retarded by the pH adjustment. In Figure 3b, the calculated droplet size distribution derived from Equation (3) using anodic collision data matches well with that obtained from dynamic light scattering (DLS), indicating the validity of the electrochemical size measurement.

The current spike can be effectively analyzed by applying a bulk electrolysis model to the decaying current [38]. The decay time of the collision spikes was primarily influenced by the size and contact area of the droplet. The kinetics of DA electrooxidation are very fast. Given its fast kinetics, the time required for the collision spike to decay was strongly correlated with the characteristics of the droplet, particularly its size and contact area with the UME surface.

Given the microscopic size of the droplets, we can reasonably predict that the mass transfer of DA within the droplet occurs at a sufficiently rapid rate compared to that of a macroelectrode system. By conducting a fitting analysis of the decay current, it was possible to derive the contact area of the droplets on the UME.

For each current decay characterized as a function of time, a meaningful comparison can be made by comparing it with the theoretical data evaluated using Equation (4).

(4) $$i\left(t\right)=i_{p}e^{-(mA/V)t}$$

The assumption made in the analysis was that the DA droplets underwent complete electrolysis and that this process occurred uniformly through a circular contact area. This assumption provides a simplified framework for the theoretical model, enabling the derivation of Equation (4), which relates the peak current ($i_{p}$) to the circular contact area (A), volume of the aqueous droplet (V), and electrolysis time (t). The mass transfer coefficient of the disk-type electrode is denoted m, and is represented by Equation (5).

(5) $$m=\frac{4D_{DA}}{\pi R_{e}}$$

$D_{DA}$ represents the diffusion coefficient of protonated dopamine. The radius of the circular contact area is represented by $R_{e}$ (i.e., $A=\pi R_{e}^{2}$). The volume of the aqueous droplet (V) can be determined using the formula $(4/3)\pi R_{d}^{3}$, where $R_{d}$, the droplet radius, is obtained from Equation (3).

**Figure 4:**
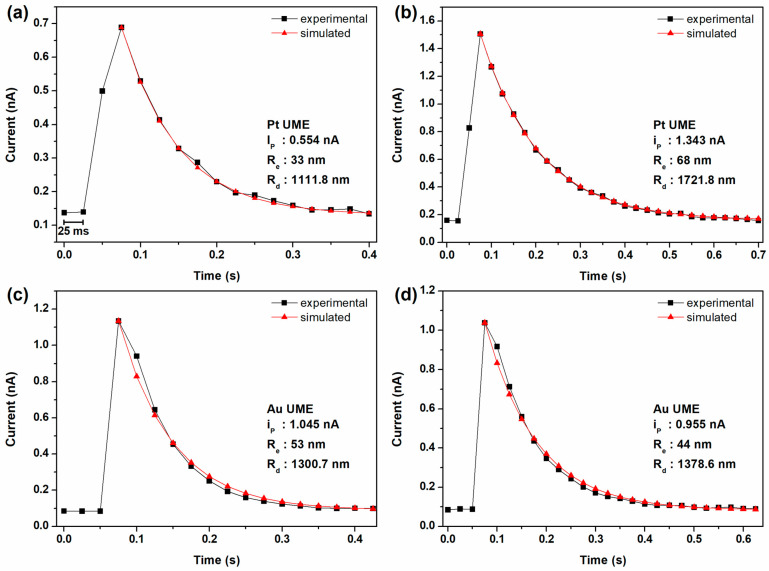
Single anodic current spikes from i–t curves with 100 mM TBAPF_6_ in DCE with emulsion droplets containing 50 mM DA and 50 mM MgSO_4_. The experiments were conducted using different working electrodes. Panels (**a**,**b**) employed Pt UME, while panels (**c**,**d**) used Au UME. The experimental data (black) and the simulated decay curves (red) are illustrated.

The experimental current spike was successfully fitted to the simulated data, as shown in Figure 4. Notably, the contact radii of the DA droplets employing the Pt UME were found to be 33 nm for a droplet with a 1.1 μm radius and 68 nm for droplet with a 1.7 μm radius. Similarly, when using Au UME, the contact radii were measured at 53 nm for a 1.3 μm radius droplet and 44 nm for a 1.4 μm radius droplet. Comparably, the contact radius–droplet radius ratio ($R_{e}$/$R_{d}$) for the DA droplets was determined to be 0.035 for the Pt UME and 0.036 for the Au UME. Notably, although the electrode materials were different, the contact areas obtained were similar.

Interestingly, compared to a previous report, the contact area ratio ($R_{e}$/$R_{d}$) was smaller than that of the ferrocyanide droplet and larger than that of the ferricyanide droplet, although every droplet was stabilized by the AOT emulsifier. Therefore, the contact area of a droplet varies depending on the redox species dissolved in the droplet. According to a previous report, the contact area may be related to the detachment properties of droplets [47]. Therefore, to study the electrochemically generated species inside a droplet, the contact area ratio was used to determine the detachment properties of the droplet.

The collision of the DA droplet with the electrode surface, which results in its adsorption and detachment, highlights an interesting aspect of the electrochemistry of nanoreactors (i.e., droplets). In single-droplet electrochemistry, a few tens of DA droplets collide with the electrode and rapidly undergo electrolysis at the surface, generating DQ droplets. Interestingly, a few tens of droplets in the electrolyte solution had zeptomolar concentrations. Some of the few tens of DQ droplets may escape from the electrode surface, followed by collision with the electrode. Based on the molar concentration of DQ droplets, the detection of DQ droplets seems nearly impossible. However, this has been measured experimentally. The unique characteristics of droplet behavior on the electrode can be used to study the reaction’s intermediate (e.g., DQ) in a single entity.

## 4. Conclusions

Dopamine is a well-known neurotransmitter delivered in microscopic vesicles. We demonstrated the electrochemistry of DA and DQ in single submicron droplets in contact with a UME. A single droplet can be studied using one of these two methods. Single droplets adsorbed onto the UME demonstrated a shift in the redox potential of DA/DQ by producing protons in a nanoscale reactor. Secondly, collisional and recollisional current spikes can be used to study the presence of DA and DQ in droplets. The effective detection of DQ is accomplished by suppressing the chemical reaction of DQ through local pH changes induced by DA oxidation. This may preserve DQ in a single vesicle if it is oxidized from DA and successive oxidation by cyclization is prohibited by a pH change. The obtained charges of the DA and DQ droplets were compared and showed that the DA droplet had a charge that was 15 times smaller. The attenuated charge may be attributed to the cyclization of DQ or fragmentation of the droplet. Overall, this study contributes to the evolving field of single-entity electrochemistry by offering insights into the behavior of single droplets in recollision and the electrochemical reactions of neurotransmitter molecules within confined microenvironments.

## Figures and Tables

**Table 1:** Previous electrochemical studies of dopamine detection with UMEs.

Electrode Material	Focus	Ref.
Carbon fiber	Scanning ion conductance microscope	[11]
Carbon fiber	Graphene-coated microelectrodes	[12]
Carbon fiber	Covalent organic framework modified electrode	[13]
Gold	Nanoporous electrode	[14]
Gold	Nanoscale electrode	[15]

**Table 2:** Gibbs transfer energies at the W–DCE interface of the ions used in this system.

Cation	$∆G_{tr,ion}^{0,water\to DCE}$ /kJ mol^−1^	Ref.	Anion	$G_{tr,ion}^{0,water\to DCE}$ /kJ mol^−1^	Ref.
H^+^	60.5	[55]	Cl^−^	45.3	[55]
NBu_4_^+^	−21.8	[56]	PF_6_^−^	8.7	[55]
Mg^2+^	122.0	[55]	SO_4_^2−^	97.6	[55]

## Data Availability

Data are contained within the article.
